# A scoping review of the uses and institutionalisation of knowledge for health policy in low- and middle-income countries

**DOI:** 10.1186/s12961-019-0522-2

**Published:** 2020-01-20

**Authors:** Adam D. Koon, Lauren Windmeyer, Maryam Bigdeli, Jodi Charles, Fadi El Jardali, Jesse Uneke, Sara Bennett

**Affiliations:** 1 Department of International Health, Johns Hopkins Bloomberg School of Public Health, Johns Hopkins University, 615 N Wolfe St, Baltimore, MD 21205 United States of America; 2 International Development Division, Abt Associates Inc, Rockville, MD United States of America; 3 Upstream USA, Oakland, CA United States of America; 4 John F Kennedy School of Government, Harvard University, Cambridge, MA United States of America; 5 World Health Organization, Geneva, Switzerland; 6 Office of Health Systems, United States Agency for International Development, Washington, D.C, United States of America; 7 American University of Beirut, Beirut, Lebanon; 8 Ebonyi State University, Abakaliki, Nigeria

**Keywords:** Evidence-based policy, Knowledge, Institutionalisation, Health policy, Low- and middle-income countries

## Abstract

There is growing interest in how different forms of knowledge can strengthen policy-making in low- and middle-income country (LMIC) health systems. Additionally, health policy and systems researchers are increasingly aware of the need to design effective institutions for supporting knowledge utilisation in LMICs. To address these interwoven agendas, this scoping review uses the Arskey and O’Malley framework to review the literature on knowledge utilisation in LMIC health systems, using eight public health and social science databases. Articles that described the process for how knowledge was used in policy-making, specified the type of knowledge used, identified actors involved (individual, organisation or professional), and were set in specific LMICs were included. A total of 53 articles, from 1999 to 2016 and representing 56 countries, were identified. The majority of articles in this review presented knowledge utilisation as utilisation of research findings, and to a lesser extent routine health system data, survey data and technical advice. Most of the articles centered on domestic public sector employees and their interactions with civil society representatives, international stakeholders or academics in utilising epistemic knowledge for policy-making in LMICs. Furthermore, nearly all of the articles identified normative dimensions of institutionalisation. While there is some evidence of how different uses and institutionalisation of knowledge can strengthen health systems, the evidence on how these processes can ultimately improve health outcomes remains unclear. Further research on the ways in which knowledge can be effectively utilised and institutionalised is needed to advance the collective understanding of health systems strengthening and enhance evidence-informed policy formulation.

## Background

Within health policy and systems research (HPSR), a growing body of literature assesses the multiple ways in which actors use various types of knowledge to inform the health policy process in low- and middle-income countries (LMICs) [1]. This is reflective of the different forms of knowledge and the processes by which these are utilised in diverse contexts and through various financial and governance arrangements [2]. Work in this area likely originated from the evidence-based policy movement, but there is a growing recognition that evidence can inform, but not determine, political decision-making [3, 4]. Much of the work in HPSR is associated with the overlapping concepts of ‘knowledge management’, ‘knowledge utilisation’ and ‘knowledge translation’, which have been criticised as being overly rational and technocratic [5]. Terminological debates aside, there remains a need to understand more about how different forms of knowledge are used, via formal and informal channels, to shape policy in ways that align with social values and societal preferences [6]. In this way, the growing body of scholarship on the use of knowledge transcends divisive strategic debates in global health [7].

Yet, key gaps persist in the knowledge requirements of government officials in fulfilling their roles [8]. For instance, it is not well understood how different forms of knowledge are used in the health policy process [9]. Little is known about how to develop institutions and processes in LMICs to support evidence use in policy and decision-making and how such institutional arrangements can support the exchange of knowledge [10]. Finally, as an aspect of health system governance, it is unclear how evidence-use contributes to health system performance or health outcomes [11].

### Types of knowledge

There is an extensive body of work seeking to define the core routine indicators that health systems should collect and analyse [12]. While such information helps to describe current health system trends, routine information may be insufficient for decision-making concerning health systems [6]. Structural elements of health policy-making are important but so too are other forms of knowledge that affect the ways leaders craft health policy. Some researchers have proposed further investigation into three types of ‘intelligence’ for health systems, as follows: (1) health systems performance, (2) context and actors, and (3) policy options [13]. Further, the existing literature on informational requirements typically focuses on empirical measures of a country’s health system (likely focused on the national level), rather than broader global evidence addressing the effectiveness of alternative health system strengthening strategies [14]. Thus, this scoping review identifies different types of knowledge useful for policy-making in LMIC health systems.

Several models have been proposed to characterise the flow of knowledge between knowledge producers (researchers) and users (policy-makers). For example, ‘researcher push’ models reflect how researchers are responsible for packaging empirical research in ways that are intelligible to policy-makers [15]. By contrast, ‘user pull’ models focus on generating demand for high quality, policy-relevant research among policy-makers [16, 17]. Another way that knowledge flows in the policy-making process is through “*linkages and exchanges*” [18] such as policy dialogues. A fourth model brings together elements of each of the previous models through large-scale knowledge translation platforms [14]. A fifth model concerns knowledge co-production, in which anticipated users of knowledge participate in the knowledge-generation process [19]. Despite research on these linkages between researchers and policy-makers, much remains unknown about how these relationships are structured [20] and the extent to which experience is transferable across contexts [21]. This scoping review pulls together these various ways in which knowledge is used in the policy process to reflect on modes of constructive engagement between researchers and policy-makers.

Researchers working in a political science tradition often argue that knowledge in its various forms serves a range of political purposes and means different things in different contexts [22]. Research outside of HPSR suggests that policy-makers value expert knowledge because it can lend authority to their predetermined policy positions and signal to others their capacity for sound decision-making, particularly in risky areas of policy [23]. Research in HPSR has further demonstrated the symbolic value of knowledge utilisation in the policy process [24] but to a limited extent in LMICs [25]. There remains a paucity of literature on the political dimensions of knowledge utilisation, particularly in LMIC health systems, where the generation and application of knowledge may differ from high-income country contexts. This review hopes to further characterise and, at least partially, fill these gaps.

### Actors, organisations and institutions

A knowledge gap also exists with regard to alternative institutional modalities for generating policy-relevant knowledge and applying this to policy-making in LMIC health systems. Some research has attempted to classify these types of institutions and the qualities that facilitate knowledge sharing [26]. Yet, research is patchy, disorganised and tends to focus more narrowly upon institutions specific to knowledge translation [27]. Moreover, little is known about how existing institutions, including think tanks, health policy and planning units, advocacy groups, and the media currently fulfill this role [6, 28]. For these institutional structures to be useful, they entail the involvement of civil society organisations and non-state actors in supporting socially constructed stewardship functions. This is akin to what Parkhurst calls the “*evidence advisory system*”, which promotes the good governance of evidence [29]. Still, much remains unknown about the character of these institutions, their arrangement in health systems and the process by which knowledge is institutionalised. This scoping review explores these themes and how they relate to the various uses of knowledge highlighted above.

### Institutionalisation

A significant gap in HPSR is not just the location or identity of institutions that produce and share knowledge, but the process by which knowledge is institutionalised for policy-making purposes. Institutionalisation is a process that emphasises stability and durability. It can be simply understood as, “*to infuse with value beyond the technical requirements of the task at hand*” [30]. According to Scott [30], “*Institutions are comprised of regulative, normative, and cultural-cognitive elements that, together with associated activities and resources, provide stability and meaning to social life*”. Institutionalisation is thus a social phenomenon that develops through shared experience and leads to path-dependent behavior.

For this study, we adapted Scott’s framework on dimensions of institutions to capture these characteristics in the health policy literature from LMICs (Table 1). First, regulative dimensions of institutionalisation are present when binding rules (i.e. laws, regulations) govern the use of knowledge for policy-making in the health sector. This is a fast and efficient way to reward or punish individuals to use specific types of knowledge for policy-making and in particular ways. Second, normative dimensions of institutionalisation are present when a value judgement, such as through formal processes of accreditation/certification or informal processes of peer feedback, have been leveraged to ensure the use of appropriate knowledge for policy-making in the health sector. This process is somewhat less fast and efficient, but it relies on social pressure to compel individuals to incorporate particular types of knowledge into policy-making and in certain ways. Third, cultural–cognitive dimensions of institutionalisation are present when knowledge use for policy-making is so commonly understood and valued that it is assumed. This process is slow but profound and difficult to change. It involves shared routines, language, protocols and beliefs about using knowledge for policy-making [30]. These three elements of institutionalisation (regulative, normative and cultural–cognitive) reflect the multifaceted nature of institutions, the dimensions of which are emphasised and explored by different disciplines.

**Table 1:** Three dimensions of institutionalisation (Adapted from Scott, 2011 [30])

	Regulative	Normative	Cultural–cognitive
Indicators	Rules, laws, sanctions	Certification, accreditation, standards/guidelines	Common beliefs, shared actions, speech, logics
Affect	Fear, guilt/innocence	Shame/honor	Certainty/confusion
Basis of legitimacy	Legally sanctioned	Morally governed	Comprehensible, culturally supported
Basis of compliance	Expedience	Social obligation	Shared (tacit) understanding
Logic	Instrumentality	Appropriateness	Orthodoxy

This scoping review thus seeks to analyse all three dimensions of institutionalisation in the HPSR literature in order to understand how actors in different contexts seek, respond to and use knowledge in the policy-making process. We utilise the well-established Arksey and O’Malley [31] framework to collate, characterise and critically appraise the existing literature in order to highlight research on knowledge and institutionalisation, and its relative merits and shortcomings. Specifically, our scoping review seeks to (1) characterise the range of research on knowledge utilisation processes, (2) the institutionalisation of these processes, and (3) the effects of these processes on health systems outcomes and health.

## Methods

This research was part of a greater endeavor called Marshalling the Evidence for Governance Contributions to Health System Performance and Health Outcomes Initiative (https://www.hfgproject.org/marshalling-evidence-health-governance/). This initiative was a collaborative enterprise involving a number of global experts and coordinated jointly by WHO and the United States Agency for International Development (USAID).

This research used scoping review methods to characterise the content of the literature and any potential gaps that require further exploration. Scoping reviews are uniquely well placed to identify what is known and unknown from vast bodies of research [31]. The scoping review methodology has been discussed in key methodological texts [32–35] and is increasingly used in HPSR (for example, see [36, 37]). This approach emphasises flexibility and demonstrates an affinity for narrative-driven summation, which, like all qualitative research, involves some degree of interpretation. The Arksey and O’Malley framework [31] is presented as an iterative, qualitative review with five distinct stages, namely (1) identifying the research question, (2) identifying relevant studies, (3) study selection, (4) charting the data, and (5) collating, summarising and reporting the results.

The research team developed the following question to drive our scoping review: “What is known from the existing health literature about how actors use and incorporate knowledge into health systems policy-making and what sorts of institutional arrangements facilitate this process in LMICs?” This question drew important distinctions related to knowledge utilisation and its institutional basis within health systems. In the context of the Marshalling the Evidence Initiative, the researchers sought to assess how these social phenomena are transformed into targeted health indicators and health system impacts.

A search of the peer-reviewed literature was conducted for original research articles that described in detail the uses of knowledge and/or their institutionalisation in health systems. Eight different social science and health databases (PubMed, Web of Science, PsychInfo, CINAHL, JSTOR, ProQuest, EBSCO, EMBASE) were searched in February and March 2017. A basic search criteria incorporated the terms (knowledge OR Evidence OR Information) AND (‘health policy’ OR ‘health systems’) And (‘low or middle income country’ OR list of relevant country names OR list of relevant country regions). This search strategy was executed by two researchers (ADK and LW), with an effective cut-off date of March 31, 2017. Articles were screened separately by both researchers based on title, abstract and then full-text. Upon full-text review, both researchers read all articles, discussed each one and came to a joint determination about which articles to include in the final review. Articles were included that describe a process for how knowledge was used in policy-making, specified the type of knowledge used, identified actors involved (individual, organisation or professional) and were set in LMICs.

**Fig. 1:**
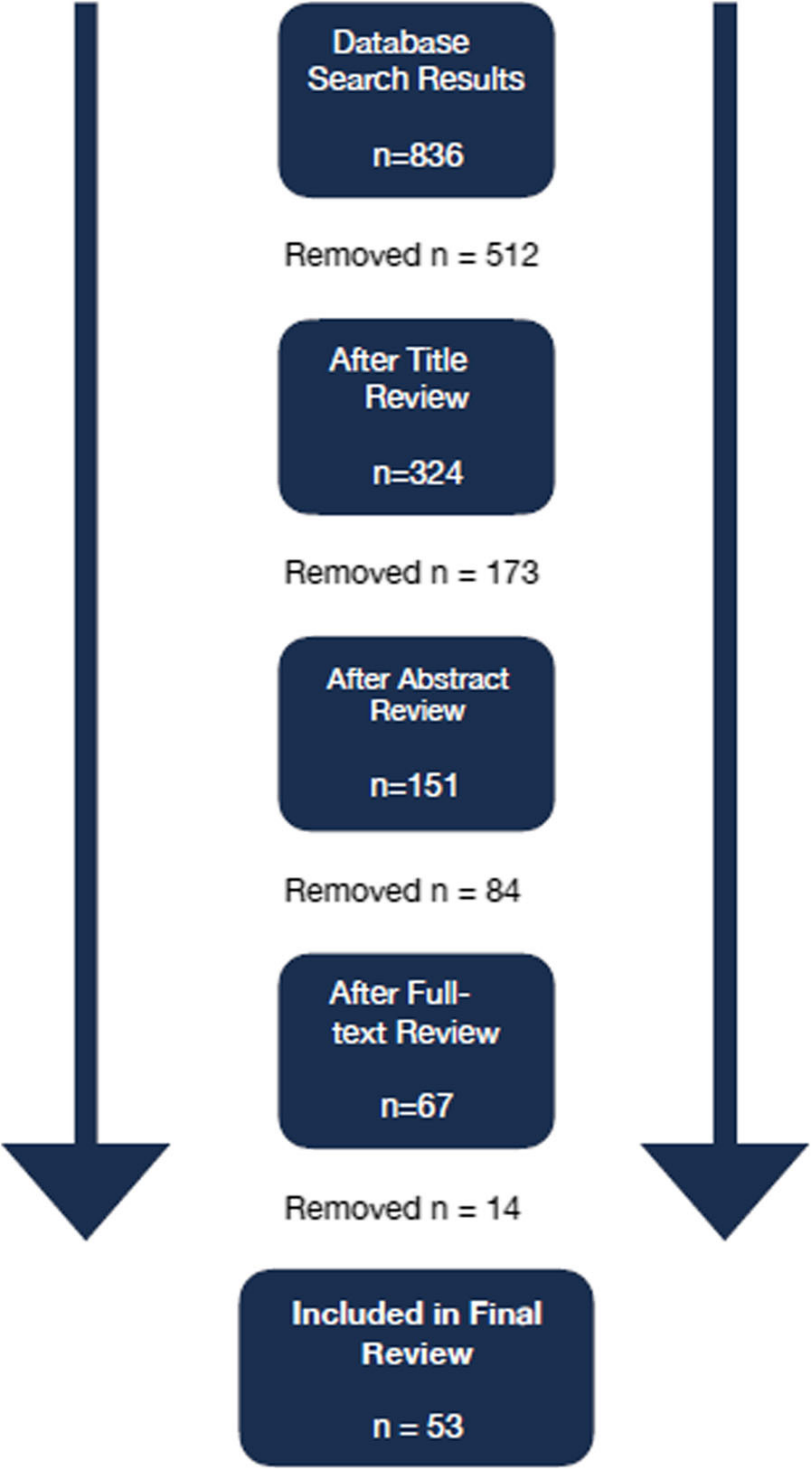
Scoping review flow diagram

Articles were excluded by ADK and LW based on their title, abstract and full-text. Title and abstract elimination were conducted in discrete rounds because of the vast array of articles and because the two reviewers wanted to ensure as broad of an interpretation of the key concepts as possible. Hence, the final collection of articles represents a shared interpretation based on clearly segmented rounds of review. This also fostered familiarity with the literature included in the full-text review. Articles were excluded that were published in a language other than English, Spanish or French and published before 1995. This date was used as the initial cut-off primarily because the authors wanted to capture some of the early work that laid the conceptual foundation of HPSR, as presented in the World Health Report 2000 [12]. Articles were also excluded if they focused on uses of knowledge outside of the health sector, focused above the nation-state or exclusively in high-income countries, and focused largely on clinical interventions, service management or procurement. In addition, all editorials and advocacy outputs were excluded. Co-authors MB, SB and JC were consulted initially for questionable exclusions and strategies for handling articles other than original research such as review articles. See Fig. 1 for an overview of the review process.

Akin to data extraction, data ‘charting’ was initiated by LW, consistent with the Arksey and O’Malley framework [31]. The charting fields were developed in consultation with all co-authors, and ADK provided support throughout the process. A master database was created that systematically collated article details, geographic location, level of analysis (national, state, district, community), urban/rural designation, actors involved, legislation, process of institutionalisation, type of knowledge used, and how governance affects health system outcomes and health impact. Yet, charting involved a degree of interpretation, appraisal and assessment on the part of the data charting researcher (LW) to classify ambiguous fields such as the process of institutionalisation and knowledge utilisation’s health outcomes/impact. ADK provided consistent advice throughout the charting process and both LW and ADK reflected on the basis of their shared interpretation. This included clarifying the charting fields, capturing information in adequate detail, and determining how to assess questionable entries.

Many research studies were initially screened based on inclusion/exclusion criteria. A total of 836 articles were returned from the initial search by researchers (ADK and LW). From these, a title review, supplemented with cursory abstract review, further narrowed the number of articles to 324. The exclusion/inclusion criteria were applied in the next round of review to all abstracts and, when necessary, a cursory full-text review. This reduced the total number of remaining entries to 151. AK and LW carefully reviewed the full-text of all articles before further narrowing down to 67. AK and LW subsequently discussed each article at length, reflecting on the inclusion/exclusion criteria and their interpretations of the phenomena under investigation. Finally, following this review of all full-text articles, 53 articles were determined to adequately include all of the study research criteria and remain in this study. See Additional file 1 for an overview of all 53 articles, which are characterised in greater detail below. See Additional file 2 for details of the search strategy.

The final stage of the scoping review process involved collating, summarising and reporting the findings. Collated articles were characterised by charting field, with emerging trends identified for multiple variables. The scope of existing knowledge was emphasised in characterising the pool of collated articles, and we identified key gaps in the literature and areas for further research on knowledge utilisation and institutionalisation.

Author reflexivity is important because interpretation and narrative summation are central to the Arksey and O’Malley scoping review framework [31]. The authors of this manuscript represent a variety of geographical locations and come from different disciplines. We are united by a common focus on HPSR as an applied problem-solving area of inquiry in global health. The study design and review process operated under the assumption that this study can contribute to strengthening the basis for policy-making in LMICs in addition to pooling a unique body of research to advance scientific inquiry in the field. Though we make no claims to objectivity, we have attempted to provide a fair and balanced account of the various strands of research and their representation in the health literature. Thus, the work bridges and embodies a plurality of ontological and epistemological positions on knowledge and research, consistent with moves towards analytical eclecticism in policy studies [38].

## Results

We found that most research was published in the last 8 years from a variety of LMICs (Table 2). Though our search start dates were from 1995 to March 2017, the earliest article to meet our search criteria was published in 1999 and the most recent was published in 2016. Relevant research articles are increasing rapidly in volume and geographic coverage over time, as follows: 1995–1999 (*n* = 1 article), 2000s (*n* = 13 articles) and 2010s (*n* = 39 articles), though this may reflect broader trends in HPSR [39]. Studies were reported from several LMICs (*n* = 56), with Uganda (*n* = 11), Nigeria (*n* = 9) and Bangladesh (*n* = 7) representing the highest number of articles. Over half of the studies focused on a single country (55%, *n* = 30), whereas 23 involved more than one country (*n* = 17 multi-country studies; *n* = 6 regional studies).

**Table 2:** Characteristics of included papers

	Total (*n*, %)
Geographic coverage	
Multiple countries	23 (45%)
Uganda	11 (21%)
Nigeria	9 (17%)
Bangladesh	7 (13%)
Others	< 7
Administrative focus	
National level	39 (74%)
Regional level	7 (13%)
District level	2 (4%)
State level	1 (2%)
Multiple levels	4 (8%)
Rural vs. urban	
Urban	47 (87%)
Rural	6 (13%)
Language	
English	52 (98%)
Spanish	1 (2%)
Source	
Original research articles	49 (92%)
Review articles	4 (8%)

Roughly half focused on a single country, using research conducted at the national level and in urban areas. The majority of studies (87%, *n* = 47) were conducted in urban areas, while only one was conducted exclusively in a rural area. Studies were located at different administrative tiers of the health system, with the majority of research conducted at the national level (*n* = 39), followed by regional (neighboring country) (*n* = 7), district (*n* = 2) and state (*n* = 1) studies, and studies that operated at multiple levels (*n* = 4). Further, 41% of the studies (*n* = 21) addressed an explicit initiative to promote and/or institutionalise an intervention aimed to promote the use of evidence in policy-making; however, of the remaining studies, it was not always clear whether such an explicit initiative existed or whether the studies were purely observational.

Nearly all of the studies were written in English (*n* = 52), while one was in Spanish. The search and selection criteria returned original research articles (*n* = 49) and review articles (*n* = 4). Research was published in a variety of public health journals (*n* = 26), including *Health Research Policy and Systems* (*n* = 9), *Health Policy and Planning* (*n* = 5), *BMC Health Services Research* (*n* = 4), *BMC Public Health* (*n* = 4) and the *International Journal of Health of Technology Assessment in Health Care* (*n* = 4).

### Types of knowledge

Different types of knowledge were used to inform policy-making in the HPSR literature (Table 3). Research was oriented around scientific knowledge (*n* = 37 articles), pragmatic skill-based (technical) knowledge (*n* = 10) or was unspecified (*n* = 10). There was a single example of deliberative value-based ethics (phronesis) that relied on principles of reflective practice, akin to auto-ethnography [40]. Research was categorised by the type of knowledge used for policy-making purposes. Just over half of the articles (*n* = 27) highlighted the use of research to inform policy-making. Many also illustrated the use of routine epidemiological or health system data (*n* = 15), survey data (*n* = 12), advice (*n* = 11), economic evaluations (*n* = 4), reports (*n* = 4), or civic participation (*n* = 4). Several articles (*n* = 10) referred to multi-faceted forms of knowledge without clearly differentiating them. The majority of research from this review presented uses of scientific knowledge as represented by research findings and, to a lesser extent, technical advice, routine health systems data and survey data.

**Table 3:** Descriptive overview

	Total (*n*, %)
Types of knowledge	
Scientific (epistemic)	37 (72%)
Technical (pragmatic, skill based)	10 (19%)
Unspecified	10 (19%)
Source of knowledge	
Research	27 (51%)
Routine data collection	15 (28%)
Survey data	12 (23%)
Advice	11 (23%)
Economic evaluation	4 (8%)
Reports	4 (8%)
Civic participation	4 (8%)
Actors	
Health officials	43 (81%)
Civil society	21 (40%)
International stakeholders	19 (36%)
Academics	17 (32%)
In-country programmes or projects	12 (25%)
Technical advisory groups	11 (21%)
Think tanks	2 (4%)
Media	2 (4%)
Unspecified	2 (4%)
Dimensions of institutionalisation	
Normative (certification, accreditation, norm)	46 (89%)
Cultural–cognitive (beliefs, axioms, scripts)	16 (30%)
Regulative (rules, laws, sanctions)	8 (15%)
Demonstrated outcomes and impacts	
Health system outcomes	24 (45%)
Health impacts	7 (13%)

Several important observations were made when analysing the types of knowledge used to support policy-making in LMICs. A little over half of the articles (*n* = 27) articulated specific examples of research being used to inform policy-making, including multiple examples of strengthening policy-makers’ capacities to incorporate research in the policy-making process in Nigeria [41–43] and research on catastrophic health expenditure being used to inform the design of a new health insurance programme in Mexico [44]. Similarly, an analysis of the policy process for the introduction of male circumcision for HIV prevention in Uganda illustrated how research (particularly randomised controlled trials) was used to inform the national policy agenda in 2007 [45]. Two multi-country studies demonstrated how efforts to enhance research capacity [46] and develop policy dialogues [47] resulted in research-informed policy-making. In this way, much of the literature included in this review focuses on the use of research as a particularly helpful form of knowledge to inform policy-making.

An interesting finding of this review is that less-structured types of knowledge, such as advice (*n* = 11) and inputs from civil society (*n* = 4), were used for policy-making purposes. The role of advice, particularly in the form of technical guidance, was pronounced in studies concerning vaccines [48, 49], health technology assessment [50–52] and pharmaceutical policy [53]. WHO seems to be well-positioned in this process as some studies focused on its ability to establish technical guidelines and convene diverse groups of stakeholders [53–56]. On the other hand, input from civil society organisations was seen as a crucial element of forming deliberative policy dialogue [57–60]. In this way, technical advice and civic participation were considered essential, and arguably overlooked, forms of knowledge for policy-making in health systems.

### Actors, organisations and institutions

A variety of actors, organisations and institutions were represented by this cohort of HPSR research. Across this literature an average of three to four categories of actors (*n* = 196 actors/53 articles) were explicitly identified in the process of knowledge utilisation. This represented a mix of organisational and institutional entities. The most frequently mentioned actors in the policy process were domestic government employees, mostly health officials (*n* = 43), civil society (*n* = 21), international stakeholders, including donors, bilateral and multilateral representatives (*n* = 19), academics (*n* = 17), in-country programmes or projects (*n* = 12), and technical advisory groups (*n* = 11). Think tanks (*n* = 2), media (*n* = 2) and unspecified actors (*n* = 2) were represented to a lesser degree. In summary, most of the articles in this review concentrate on domestic public sector employees and their interactions with civil society representatives, international stakeholders or academics in utilising scientific knowledge for policy-making in LMICs.

In general, articles were characterised by an array of actors, including domestic government officials, civil society, international stakeholders and academic researchers. The largest number of different types of stakeholders (*n* = 10) engaged in knowledge translation for policy-making were identified by multiple articles from an ongoing research effort in Nigeria [42, 43, 61]. Most of the articles (*n* = 43) focused on domestic governments, a stated emphasis of this review. Many articles (*n* = 21) included civil society participation, usually in the form of non-governmental organisations [62], but also directly with communities themselves [63]. International stakeholders (*n* = 19) and academics (*n* = 17) were also well-represented in the literature. Surprisingly, no study illustrated the various uses of knowledge among the four groupings of actors simultaneously (domestic government officials, international stakeholders, civil society and academics). Just three articles explicitly mentioned knowledge exchanges among government officials, international stakeholders and academics [45, 46, 51].

The most frequent interaction among these four entities were studies that highlighted exchanges between domestic governments, international stakeholders and civil society (*n* = 6). This included research on integrated community case management in Malawi [64], coordination of policy dialogue in Guinea [62], aid coordination and policy formulation in South Sudan [63], policy dialogues in three West African countries [47], Global Fund financing in Brazil [65], and the policy process for maternal health in Ghana [66]. In this way, the body of research suggests that it is widely acknowledged that many actors are involved in the process of exchanging knowledge in LMICs, with the engagement of civil society, international stakeholders and domestic government officials central to this dynamic.

While some articles highlighted the role of key individuals in positions of authority, most articles did not distinguish between individual actors, organisations and institutions. Instead, most research focused at the organisational level. The lone exception to this was a multi-country effort to strengthen individual, organisational and institutional capacity to use research for policy-making by Hawkes et al. [67]. The authors noted, however, that none of their study countries were fully engaged in institutional capacity development despite its widely acknowledged importance for sustainability. Rather, the authors posited that *“developing individual and organizational capacity is a pre-requisite for seeing long-term institutional change”* [67]. Therefore, it is plausible that processes of knowledge use in the study countries might be heading towards full institutionalisation, but the groundwork has yet to be sufficiently established to build regulative, normative and cultural–cognitive platforms to achieve this.

### Institutionalisation

Different dimensions of institutionalised knowledge use point to emerging themes in HPSR. The vast majority of articles identified normative dimensions of institutionalisation (*n* = 46). Cultural–cognitive dimensions of institutionalisation (*n* = 16 articles) were represented more frequently than regulative dimensions (*n* = 8 articles). In most of the articles represented in this review, institutionalisation occurred through a process of strengthening norms around the use of knowledge in policy-making. This occurred in informal ways by reducing the barriers between knowledge producers (researchers) and knowledge users (policy-makers), and occasionally by formal mechanisms such as developing processes akin to accreditation or certification. Another frequent mechanism by which normative dimensions of institutionalisation of knowledge use were characterised was somewhere between these informal and formal processes. This is illustrated by articles that referred to the creation of technical committees or government programmes such as health technology assessment programmes. These were classified as normative dimensions because, frequently, the articles failed to mention whether or not the recommendations of technical committees or health technology assessment programmes were legally binding. Instead, these appeared to be institutions established to facilitate exchange between researchers and policy-makers, whose recommendations occupied a privileged position in policy decision-making in much the same way as guidelines functioned to establish norms through certification bodies. While indicators of normative institutionalisation were occasionally mentioned through processes of accreditation or certification [56, 68], many articles reported efforts to strengthen norms around knowledge use. For example, the literature appears largely focused on creating an ideal environment for facilitating knowledge transfer, exchange and dialogue to better inform policy-making.

Legislation was explicitly mentioned in few (*n* = 5) articles, though it was implied in additional (*n* = 3) articles. Three review articles [25, 49, 54] reflected on regulative aspects of institutionalisation of knowledge use and a further two research articles [69, 70] addressed the evolution of regulatory mechanisms responsible for knowledge transfer. Still, there appears to be a gap in the health literature on regulative forms of institutionalisation that adhere to binding rules and structured incentives for the purpose of expedient knowledge transfer. Further evidence concerning this binding, but efficient, form of institutionalisation of knowledge use for decision-making – how it can be enacted and how effective it might be in LMIC settings – is needed.

Cultural–cognitive dimensions of institutionalisation of knowledge use were represented more frequently than regulative dimensions, but less so than normative dimensions. Notably, cultural–cognitive institutionalisation was never fully documented in any of the studies, yet aspects of it were present in studies on citizen involvement in the health policy process in Brazil [60], in three case studies of non-governmental organisation involvement in policy-making [71], and in creating effective policy dialogues in West Africa [47].

Research on the impact of the Fogarty International Center [46] is illustrative of the ways in which cultural–cognitive institutionalisation for knowledge use can surface. Through training, epistemic communities and intergenerational linkages were created around knowledge use for policy-making. In fact, features of cultural–cognitive institutionalisation are present in the original conception of epistemic communities [72], in which individuals are bound by a common understanding of the world around them and how to interact with it. Morevoer, in the case of the Fogarty International Center, many of the trainees moved into decision-making positions as their careers advanced. In doing so, they carried the beliefs, practices and ways of interacting with their colleagues that were shaped by their training experience, which included uses of knowledge in policy-making. This example illustrates the incremental, durable and covert quality of cultural–cognitive institutionalisation.

In fact, it could be argued that most of the policy dialogue literature focuses indirectly on cultural–cognitive institutionalisation, whereby individuals interact in order to develop a common understanding about how to use knowledge for policy-making. This also could be characterised as depicting normative dimensions of institutionalisation of knowledge use, in so much as social pressure induces individuals to behave in a particular way. This is also true for studies that were conducted at regional level [54, 55, 73–75], which seek to develop a common understanding and establish modes of practice that can be shared across similar country contexts. For this reason, we considered these articles to illustrate both normative and cultural–cognitive types of institutionalisation.

The boundaries between these three dimensions of institutionalising knowledge for policy-making are not always clear. Vaccine advisory committees [48, 49, 76], health technology assessment programmes [50–52, 70] and drug policy [53, 68, 69, 77] are established with normative aims, meanih that the recommendations are not binding but rather provide an indication of how policy-makers should behave. However, they appear at times to have a regulative (legislative) basis for their formation, even if their recommendations are not binding. Similarly, a great deal of research on policy dialogues is largely normative in nature, but also overlapping to a limited extent with the cultural–cognitive processes of institutionalisation of knowledge use (as mentioned above). There was no specific example of research (i.e. discourse analysis, ethnography, deconstruction) conducted on cultural–cognitive dynamics; however, virtually all of the policy dialogue and policy exchange literature seems to imply that some form of culture–cognitive institutionalisation of knowledge use is a goal [43, 47, 58, 62, 78].

### Health system outcomes and health impacts

Nearly half of the articles reviewed (*n* = 24) described health system outcomes of varying specificity, though mostly policy formulation, through the establishment of guidelines, standards or broader organisational development measures. In contrast, there were few articles (*n* = 7) that described health impacts. While there is evidence of how different uses and institutionalisation of knowledge can strengthen health systems, the evidence on how these processes can impact health remains unclear.

Both health system outcomes and health impacts were qualitatively reported in vague detail and documented using process-oriented indicators and outcomes. Still, while there were a few examples of knowledge utilisation, particularly research findings and routine health system data informing policy-making, the majority of research included in this review did not document health system outcomes and health impacts. Moreover, virtually all of the research followed a similar form whereby it is documented how knowledge informs policy and health system improvements, and then health impacts are claimed to be linked. There were no experimental studies isolating systems of knowledge usage to attribute their impacts in a rigorous manner. Moreover, the ability of governance research to assess these types of effects remains debatable.

Outcomes related to health systems performance were reported for several studies. This included the incorporation of research findings into national-level policy and strategy documents [46], the creation of new state agencies or units [50, 51, 70, 79, 80], and agenda-setting for the policy process [40, 66]. The utilisation of knowledge to improve financial protection was illustrated in research from Mexico, which resulted in a reduction in out-of-pocket expenditure [44], and research from Colombia that noted a decline in spending for oncological treatment by users [77]. Some articles focused on deliberative modes of policy governance through engagement with civil society organisations that resulted in better representation and accountability [60, 65, 81]. Additionally, multple articles reflected on the use of research and routine system information to improve access to essential medicines and other pharmaceuticals [50, 53, 61, 68, 69, 77, 82]. Finally, knowledge utilisation was understood to enhance the quality of service delivery in research on integrated community case management in Malawi [64], non-communicable disease service delivery in five Asian countries [80], multiple primary care services in Nigeria [41], and male circumcision for HIV prevention in Uganda [45]. In this way, the review identified numerous studies that could loosely be characterised as corresponding to health system improvements.

Health impacts of knowledge use and institutionalisation were reported for a few articles with varying levels of specificity. Some research suggested that health impacts were achieved indirectly through health system improvements, such as improved malaria treatment in Uganda [53], reduced catastrophic expenditures in Mexico [44], improved drug availability in Tanzania [68] and increased access to emergency contraception in multiple countries [71], though these impacts were asserted rather than measured. There were just three studies that explicitly mentioned indicators of health impacts, including reductions in prevalence of hypertension in Cambodia and diabetes in Fiji [80], reduced alcohol consumption, tobacco use and increased exercise in Thailand [79], and a reduction in tuberculosis prevalence in Brazil [65]. Thus, a very small body of literature suggests any health impacts related to increased knowledge use and institutionalisation for policy-making in LMIC health systems.

## Discussion

This review found growing evidence on the multiple uses and institutionalisation of knowledge for policy-making as well as limited evidence on the corresponding health system outcomes and health impacts of these processes in LMIC health systems. A total of 53 articles, from 1999 to 2016 and representing 56 countries, were identified. The majority of articles in this review used research findings and (to a lesser extent) technical advice, routine health system data and survey data to inform policy-making. Most of the articles in this review centered on domestic public-sector employees and their interactions with civil society representatives, international stakeholders or academics. There was little evidence about how think tanks and the media contribute to this process in LMICs. Nearly all of the articles identified normative dimensions of institutionalisation of knowledge use and a few reflected on cognitive–cultural elements. There were few articles that provided examples of regulative institutionalisation of knowledge use and much remains unknown about the role of legislation in facilitating this process. While there remains some evidence of how different uses and institutionalisation of knowledge can strengthen health systems, the evidence on whether these processes alone can generate health impacts remains unclear. Furthermore, it could be argued that measuring the health impacts of complex improvements in governance may be cost prohibitive and unnecessary.

This review suggests that institutionalisation of knowledge for health policy-making in LMICs is an emerging area of interest for HPSR scholars. This likely reflects larger trends in the evolution of the field of HPSR, where research on knowledge utilisation has helped to expand and redefine traditional disciplinary boundaries [39]. While the exact nature of institutionalisation of evidence use is still poorly understood in LMIC health systems, there is clearly a need to devote more research and attention to furthering this line of inquiry. This extends to institutionalisation of a variety of forms of knowledge that have been the focus of recent research not included in this review such as efforts to instutionalise national health accounts [83] and health system strengthening strategies [84, 85]. Refinement of existing frameworks to understand the process, the politics surrounding policy design, and long-term financing strategies to ensure sustainability are all of paramount importance if the wealth of various types of knowledge are to be harnessed to inform policy deliberation and debate in LMICs.

Recent research in this area (and after our seach cut-off) provides an idea of how process-oriented research on institutionalisation of knowledge use in LMIC health systems might be conducted. One is a body of work devoted to the formation of institutionalised structures for knowledge-informed policy-making in Burkina Faso [86–88]. This work is notable for the extent to which it implicitly addresses all three dimensions of institutionalisation (regulative, normative and cultural–cognitive) as well as its practical implications for health system development. While it does not describe health system outcomes or health impacts, it does provide an unusually detailed view of institutionalisation as a dynamic social process. This finding is also shared by a recent policy and institutional analysis of a national knowledge platform in India, which carefully documents the political enterprise of institutionalising knowledge translation [89]. Further research should be conducted to develop existing frameworks and reflect on how processes of institutionalisation develop over time in different socio-political contexts.

The literature connecting knowledge use to health system outcomes and health impacts remains vague. For example, though alcohol consumption and tobacco use in youth dropped over the first few years of the Thai Health Promotion Foundation (ThaiHealth), it is difficult to determine the extent to which the results can be directly attributed to the process of knowledge use and institutionalisation [79, 90]. At a minimum, other sociopolitical conditions likely played a role in reducing harmful behaviors among Thai youth. Thus, it seems that the evidence of health impacts related to knowledge use and institutionalisation is at best weak or underdeveloped.

Measuring health system outcomes seems to be more tractable because of its focus on process-level indicators. Arguably, health impacts are more difficult because the analytic focus blurs incommensurable research paradigms and also shifts from dynamic macro-level considerations to narrow individual-level biological changes. Some social science scholars argue that the principles of inquiry for social phenomena are always inadequate to investigate the causal features of the natural world [91]. For these scholars, context, judgement and timing render human behavior unpredictable; therefore, complex social processes such as knowledge utilisation and institutionalisation will always yield incommensurable and insufficient causal explanations for biological processes [92]. This is perhaps one reason for the paucity of research on health impacts. Another possible reason is that it either is too difficult to accomplish from a research standpoint or, more simply, little attention has been paid to it until relatively recently.

Despite the contributions of this review, there remain several limitations and opportunities for further thinking about the study of both knowledge and institutionalisation in HPSR. First, the abstract nature of both knowledge and institutionalisation proved difficult to reconcile in a systematic way. For example, institutionalisation is a complicated process that involves a degree of nuance that was difficult to adequately capture in the charting stage of the review. Similarly, the outcomes and impacts of knowledge utilisation were less clear and not readily identifiable. The inclusion and exclusion criteria were such that they resulted in title review of diverse articles, which may have led to some articles being unfairly excluded. This was offset to some extent by the use of multiple reviewers, but the boundaries of knowledge utilisation remain fuzzy at best. Another limitation was that only literature with a health sector focus was reviewed and salient research on the policy process might exist in other social sectors that remain outside the purview of our original research question. Nonetheless, this research would further our understanding of the social phenomena in question.

## Conclusion

This scoping review identified a number of ways in which knowledge has been used and institutionalised for policy-making in LMICs. While there is relatively little known about improved knowledge utilisation and the effects of institutionalisation on health system outcomes and health impacts, research in this area may prove unnecessary and impractial. Instead, further efforts should be made to understand alternative forms of knowledge and how they can be used or institutionalised for policy-making in LMIC health systems. In this way, experience acquired through national and sub-national experimentation can be shared, accelerating health system strengthening endeavors globally and contributing to a healthier planet.

## Supplementary information

**Additional file 1.** Charting Database: Details for each article included in the review.

**Additional file 2.** Search Strategy: A detailed summary of the search terms used to conduct the review.

## Data Availability

The datasets used and/or analysed during the current study are available from the corresponding author on reasonable request.

## Abbreviations

HPSR: Health policy and systems research
LMICs: Low- and middle-income countries
