# Supplementary material for: A scoping review of the uses and institutionalisation of knowledge for health policy in low- and middle-income countries
Source: Health Res Policy Syst. 2020 Jan 20;18:7. doi: 10.1186/s12961-019-0522-2 (PMC6971874; doi:10.1186/s12961-019-0522-2)
Supplement: Supplementary file 2 — Additional file 2. Search Strategy: A detailed summary of the search terms used to conduct the review. [file 12961_2019_522_MOESM2_ESM.docx]

Search Strategy

Results

ProQuest: 146

Embase: 96

JSTOR: 76

EBESCO: 84

PubMed: 162

Web of Science: 206

PsychInfo: 21

CINAHL: 45

Total extraction: 836

Total after title deletion: 324

Total after abstract deletion: 151

Total after full text deletion: 67 (53 after consultation)

Search Terms

**ProQuest**: Standard search, listed below.

**Embase**:

"low or middle income country" OR "subsaharan africa" OR "sub saharan africa" OR "low income country" OR "low income countries" OR "developing country" OR "developing countries" OR "middle income country" OR "middle income countries" OR Africa OR Asia OR Caribbean OR West Indies OR South America OR Latin America OR Central America

**JSTOR**:

Search 1: "low or middle income country" OR "low income country" OR "low income countries" OR "developing country" OR "developing countries"

Search 2: "subsaharan africa" OR "sub saharan africa" or "middle income country" OR "middle income countries"

Search 3: (Africa tia OR Asia tia OR Caribbean tia OR West Indies tia OR South America tia OR Latin America tia OR Central America))

*Note: JSTOR would not allow me to search more than a few terms at a time in Field 3, so I conducted the three separate searchers listed above.*

**EBESCO**:

Standard search but removed the “all fields” bracket whenever it appeared.

**PubMed:**

Standard search with “institutional*” in Field 1 and Field 2

**Web of Science:**

Standard search

**PsychInfo:**

Standard search

**CINAHL:**

Standard search

Standard Search:

Selected “search abstracts” when applicable.

Line 1:“Knowledge” or “evidence” or information”

Line 2: “health policy” OR “health systems”

Line 3: "low or middle income country" OR "subsaharan africa" OR "sub saharan africa" OR "emerging country" [all fields] OR "emerging countries" [all fields] OR "emerging nation" [all fields] OR "emerging nations" [all fields] OR "emerging population" [all fields] OR "emerging populations" [all fields] OR "developing country" [tia] OR "developing countries" [tia] OR "developing nation" [tia] OR "developing nations" [tia] OR "developing population" [tia] OR "developing populations" [tia] OR "developing world" [tia] OR "less developed country" [tia] OR "less developed countries" [tia] OR "less developed nation" [tia] OR "less developed nations" [tia] OR "less developed population" [tia] OR "less developed populations" [tia] OR "less developed world" [tia] OR "lesser developed country" [tia] OR "lesser developed countries" [tia] OR "lesser developed nation" [tia] OR "lesser developed nations" [tia] OR "lesser developed population" [tia] OR "lesser developed populations" [tia] OR "lesser developed world" [tia] OR "under developed country" [tia] OR "under developed countries" [tia] OR "under developed nation" [tia] OR "under developed nations" [tia] OR "under developed population" [tia] OR "under developed populations" [tia] OR "under developed world" [tia] OR "underdeveloped country" [tia] OR "underdeveloped countries" [tia] OR "underdeveloped nation" [tia] OR "underdeveloped nations" [tia] OR "underdeveloped population" [tia] OR "underdeveloped populations" [tia] OR "underdeveloped world" [tia] OR "middle income country" [tia] OR "middle income countries" [tia] OR "middle income nation" [tia] OR "middle income nations" [tia] OR "middle income population" [tia] OR "middle income populations" [tia] OR "low income country" [tia] OR "low income countries" [tia] OR "low income nation" [tia] OR "low income nations" [tia] OR "low income population" [tia] OR "low income populations" [tia] OR "lower income country" [tia] OR "lower income countries" [tia] OR "lower income nation" [tia] OR "lower income nations" [tia] OR "lower income population" [tia] OR "lower income populations" [tia] OR "undeserved country" [tia] OR "undeserved countries" [tia] OR "undeserved nation" [tia] OR "undeserved nations" [tia] OR "undeserved population" [tia] OR "undeserved populations" [tia] OR "undeserved world" [tia] OR "under served country" [tia] OR "under served countries" [tia] OR "under served nation" [tia] OR "under served nations" [tia] OR "under served population" [tia] OR "under served populations" [tia] OR "under served world" [tia] OR "deprived country" [tia] OR "deprived countries" [tia] OR "deprived nation" [tia] OR "deprived nations" [tia] OR "deprived population" [tia] OR "deprived populations" [tia] OR "deprived world" [tia] OR "poor country" [tia] OR "poor countries" [tia] OR "poor nation" [tia] OR "poor nations" [tia] OR "poor population" [tia] OR "poor populations" [tia] OR "poor world" [tia] OR "poorer country" [tia] OR "poorer countries" [tia] OR "poorer nation" [tia] OR "poorer nations" [tia] OR "poorer population" [tia] OR "poorer populations" [tia] OR "poorer world" [tia] OR "developing economy" [tia] OR "developing economies" [tia] OR "less developed economy" [tia] OR "less developed economies" [tia] OR "lesser developed economy" [tia] OR "lesser developed economies" [tia] OR "under developed economy" [tia] OR "under developed economies" [tia] OR "underdeveloped economy" [tia] OR "underdeveloped economies" [tia] OR "middle income economy" [tia] OR "middle income economies" [tia] OR "low income economy" [tia] OR "low income economies" [tia] OR "lower income economy" [tia] OR "lower income economies" [tia] OR "low gdp" [tia] OR "low gip" [tia] OR "low gross domestic" [tia] OR "low gross national" [tia] OR "lower gdp" [tia] OR "lower gip" [tia] OR "lower gross domestic" [tia] OR "lower gross national" [tia] OR laic[tia] OR laics[tia] OR "third world" [tia] OR "lams country" [tia] OR "lams countries" [tia] OR "transitional country" [tia] OR "transitional countries" [tia] OR Africa[tia] OR Asia[tia] OR Caribbean[tia] OR West Indies[tia] OR South America[tia] OR Latin America[tia] OR Central America[tia] OR "Atlantic Islands" [tia] OR "Commonwealth of Independent States" [tia] OR "Pacific Islands" [tia] OR "Indian Ocean Islands" [tia] OR "Eastern Europe" [tia] OR Afghanistan[tia] OR Albania[tia] OR Algeria[tia] OR Angola[tia] OR Armenia[tia] OR Armenian[tia] OR Azerbaijan[tia] OR Bangladesh[tia] OR Benin[tia] OR Byelarus[tia] OR Byelorussian[tia] OR Belarus[tia] OR belorussians[tia] OR belorussian[tia] OR Belize[tia] OR Bhutan[tia] OR Bolivia[tia] OR Bosnia[tia] OR Herzegovina[tia] OR herzegovina[tia] OR Botswana[tia] OR Brasil[tia] OR Brazil[tia] OR Bulgaria[tia] OR Burkina Faso[tia] OR Burkina tasso[tia] OR Upper Volta[tia] OR burundi[tia] OR burundi[tia] OR Cambodia[tia] OR Khmer Republic[tia] OR Kampuchea[tia] OR Cameroon[tia] OR cameroon[tia] OR Cameron[tia] OR Cape Verde[tia] OR Central African Republic[tia] OR Chad[tia] OR China[tia] OR Colombia[tia] OR Comoros[tia] OR Comoro Islands[tia] OR comoros[tia] OR Mayotte[tia] OR Congo[tia] OR Zaire[tia] OR Costa Rica[tia] OR Cote d'Ivoire[tia] OR Ivory Coast[tia] OR Cuba[tia] OR Czechoslovakia[tia] OR Slovakia[tia] OR Djibouti[tia] OR French somalians[tia] OR Dominica[tia] OR Dominican Republic[tia] OR East Timor[tia] OR East Timur[tia] OR Timor Leste[tia] OR Ecuador[tia] OR Egypt[tia] OR El Salvador[tia] OR Eritrea[tia] OR Ethiopia[tia] OR Fiji[tia] OR Gabon[tia] OR cantonese Republic[tia] OR Gambia[tia] OR Gaza[tia] OR Georgia Republic[tia] OR Georgian Republic[tia] OR Ghana[tia] OR Gold Coast[tia] OR Grenada[tia] OR Guatemala[tia] OR guinean[tia] OR Guiana[tia] OR Guyana[tia] OR Haiti[tia] OR Honduras[tia] OR India[tia] OR Maldives[tia] OR Indonesia[tia] OR Iran[tia] OR Iraq[tia] OR Jamaica[tia] OR Jordan[tia] OR Kazakhstan[tia] OR Kazakh[tia] OR Kenya[tia] OR Kiribati[tia] OR Korea[tia] OR Kosovo[tia] OR Kyrgyzstan[tia] OR kirghizes[tia] OR Kyrgyz Republic[tia] OR Kirghiz[tia] OR kyrgyzstan[tia] OR "Lao pur" [tia] OR Laos[tia] OR Lebanon[tia] OR Lesotho[tia] OR basutos[tia] OR Liberia[tia] OR Libya[tia] OR Macedonia[tia] OR Madagascar[tia] OR Malagasy Republic[tia] OR Malaysia[tia] OR Malaya[tia] OR Malay[tia] OR Sabah[tia] OR Sarawak[tia] OR Malawi[tia] OR Nyasaland[tia] OR Mali[tia] OR Marshall Islands[tia] OR Mauritania[tia] OR Mauritius[tia] OR paralegal Islands[tia] OR "Melanesia" [tia] OR Mexico[tia] OR Micronesia[tia] OR Middle East[tia] OR Moldova[tia] OR moldova[tia] OR moldavian[tia] OR Mongolia[tia] OR Montenegro[tia] OR Morocco[tia] OR iwai[tia] OR Mozambique[tia] OR Myanmar[tia] OR Myanma[tia] OR Burma[tia] OR namibia[tia] OR Nepal[tia] OR Nicaragua[tia] OR Niger[tia] OR Nigeria[tia] OR Muscat[tia] OR Pakistan[tia] OR Palau[tia] OR Palestine[tia] OR panorama[tia] OR Paraguay[tia] OR Peru[tia] OR Philippines[tia] OR Philipines[tia] OR philippines[tia] OR philippines[tia] OR Romania[tia] OR Rumania[tia] OR Roumania[tia] OR Rwanda[tia] OR Ruanda[tia] OR Saint Kitts[tia] OR St Kitts[tia] OR Nevis[tia] OR Saint Lucia[tia] OR St Lucia[tia] OR Saint Vincent[tia] OR St Vincent[tia] OR Grenadines[tia] OR Samoa[tia] OR Samoan Islands[tia] OR Navigator Island[tia] OR Navigator Islands[tia] OR sago Tome[tia] OR Senegal[tia] OR Serbia[tia] OR Montenegro[tia] OR Sierra Leone[tia] OR Sri Lanka[tia] OR Ceylon[tia] OR Solomon Islands[tia] OR Somalia[tia] OR Sudan[tia] OR suriname[tia] OR surinam[tia] OR Swaziland[tia] OR Syria[tia] OR Syrian[tia] OR Tajikistan[tia] OR tadzhikistan[tia] OR tajikistan[tia] OR tadzhikistan[tia] OR Tanzania[tia] OR Thailand[tia] OR Togo[tia] OR Togolese Republic[tia] OR Tonga[tia] OR Tunisia[tia] OR Turkey[tia] OR Turkmenistan[tia] OR Turkmen[tia] OR Tuvalu[tia] OR Uganda[tia] OR Ukraine[tia] OR Uzbekistan[tia] OR Uzbek OR Vanuatu[tia] OR New Hebrides[tia] OR vietnam[tia] OR Viet noam[tia] OR West Bank[tia] OR Yemen[tia] OR Yugoslavia[tia] OR Zambia[tia] OR Zimbabwe[tia] OR Rhodesia[tia] OR Developing Countries[nesh] OR Africa[nesh:noels] OR Africa, Northern[nesh:noels] OR Africa South of the Sahara[nesh:noels] OR Africa, Central[nesh:noels] OR Africa, Eastern[nesh:noels] OR Africa, Southern[nesh:noels] OR Africa, Western[nesh:noels] OR Asia[nesh:noels] OR Asia, Central[nesh:noels] OR Asia, Southeastern[nesh:noels] OR Asia, Western[nesh:noels] OR Caribbean Region[nesh:noels] OR West Indies[nesh:noels] OR South America[nesh:noels] OR Latin America[nesh:noels] OR Central America[nesh:noels] OR "Atlantic Islands" [nesh:noels] OR "Commonwealth of Independent States" [nesh:noels] OR "Pacific Islands" [nesh:noels] OR "Indian Ocean Islands" [nesh:noels] OR "Europe, Eastern" [nesh:noels] OR Afghanistan[nesh] OR Albania[nesh] OR Algeria[nesh] OR American Samoa[nesh] OR Angola[nesh] OR Armenia[nesh] OR Azerbaijan[nesh] OR "Baltic States" [nesh] OR Bangladesh[nesh] OR Benin[nesh] OR "Republic of Belarus" [nesh] OR Belize[nesh] OR Bhutan[nesh] OR Bolivia[nesh] OR Bosnia-Herzegovina[nesh] OR Botswana[nesh] OR Brazil[nesh] OR Bulgaria[nesh] OR Burkina Faso[nesh] OR burundi[nesh] OR Cambodia[nesh] OR Cameroon[nesh] OR Cape Verde[nesh] OR Central African Republic[nesh] OR Chad[nesh] OR China[nesh] OR Colombia[nesh] OR Comoros[nesh] OR Congo[nesh] OR Costa Rica[nesh] OR Cote d'Ivoire[nesh] OR Cuba[nesh] OR Czechoslovakia[nesh] OR Slovakia[nesh] OR Djibouti[nesh] OR "Democratic Republic of the Congo" [nesh] OR "Democratic People's Republic of Korea" [nesh] OR Dominica[nesh] OR Dominican Republic[nesh] OR East Timor[nesh] OR Ecuador[nesh] OR Egypt[nesh] OR El Salvador[nesh] OR Eritrea[nesh] OR Ethiopia[nesh] OR Fiji[nesh] OR "French Guiana" [nesh] OR Gabon[nesh] OR Gambia[nesh] OR "Georgia (Republic)" [nesh] OR Ghana[nesh] OR Grenada[nesh] OR Guatemala[nesh] OR guinean[nesh] OR guinean-nassau[nesh] OR Guyana[nesh] OR Haiti[nesh] OR Honduras[nesh] OR "Independent State of Samoa" [nesh] OR India[nesh] OR Indonesia[nesh] OR Iran[nesh] OR Iraq[nesh] OR Jamaica[nesh] OR Jordan[nesh] OR Kazakhstan[nesh] OR Kenya[nesh] OR Korea[nesh] OR Kyrgyzstan[nesh] OR Laos[nesh] OR Lebanon[nesh] OR Lesotho[nesh] OR Liberia[nesh] OR Libya[nesh] OR "Macedonia (Republic)" [nesh] OR Madagascar[nesh] OR Malawi[nesh] OR Malaysia[nesh] OR Mali[nesh] OR Mauritania[nesh] OR Mauritius[nesh] OR "Melanesia" [nesh] OR Mexico[nesh] OR Micronesia[nesh] OR Middle East[nesh:noels] OR Moldova[nesh] OR Mongolia[nesh] OR Montenegro[nesh] OR Morocco[nesh] OR Mozambique[nesh] OR Myanmar[nesh] OR namibia[nesh] OR Nepal[nesh] OR Nicaragua[nesh] OR Niger[nesh] OR Nigeria[nesh] OR Pakistan[nesh] OR Palau[nesh] OR panorama[nesh] OR Papua New guinean[nesh] OR Paraguay[nesh] OR Peru[nesh] OR Philippines[nesh] OR "Republic of Korea" [nesh] OR Romania[nesh] OR Rwanda[nesh] OR Saint Lucia[nesh] OR "Saint Vincent and the Grenadines" [nesh] OR Samoa[nesh] OR Senegal[nesh] OR Serbia[nesh] OR Montenegro[nesh] OR Sierra Leone[nesh] OR Sri Lanka[nesh] OR Somalia[nesh] OR South Africa[nesh] OR Sudan[nesh] OR suriname[nesh] OR Swaziland[nesh] OR Syria[nesh] OR Tajikistan[nesh] OR Tanzania[nesh] OR Thailand[nesh] OR Togo[nesh] OR Tonga[nesh] OR Tunisia[nesh] OR Turkey[nesh] OR Turkmenistan[nesh] OR Uganda[nesh] OR Ukraine[nesh] OR Uzbekistan[nesh] OR Vanuatu[nesh] OR vietnam[nesh] OR Yemen[nesh] OR Yugoslavia[nesh] OR Zambia[nesh] OR Zimbabwe[nesh] OR "Southern African Development Community" [all fields] OR "East African Community " [all fields] OR "West African Health Organisation" [all fields]
